# Demographic, psychosocial and clinical factors associated with postpartum depression in Kenyan women

**DOI:** 10.1186/s12888-018-1904-7

**Published:** 2018-10-01

**Authors:** Linnet Ongeri, Valentine Wanga, Phelgona Otieno, Jane Mbui, Elizabeth Juma, Ann Vander Stoep, Muthoni Mathai

**Affiliations:** 1 Kenya Medical Research Institute, P.O. Box 54840 00200, Mbagathi Road, Nairobi, Kenya; 2 University of Washington, Jefferson St. Seattle WA 98104, Nairobi, 908 Kenya; 3 University of Nairobi, P.O. Box 30197, Off Ngong Road, Nairobi, Kenya

**Keywords:** Postpartum depression, Antenatal depression, Psychosocial risk factors, Edinburgh postpartum depression scale

## Abstract

**Background:**

Few longitudinal studies have examined associations between risk factors during pregnancy and mental health outcomes during the postpartum period. We used a cohort study design to estimate the prevalence, incidence and correlates of significant postpartum depressive symptoms in Kenyan women.

**Methods:**

We recruited adult women residing in an urban, resource-poor setting and attending maternal and child health clinics in two public hospitals in Nairobi, Kenya. A translated Kiswahili Edinburgh Postpartum Depression Scale was used to screen for depressive symptoms at baseline assessment in the 3rd trimester and follow up assessment at 6–10 weeks postpartum. Information was collected on potential demographic, psychosocial and clinical risk variables. Potential risk factors for postpartum depression were evaluated using multivariate logistic regression analysis.

**Results:**

Out of the 171 women who were followed up at 6–10 weeks postpartum, 18.7% (95% CI: 13.3–25.5) were found to have postpartum depression using an EPDS cut off of 10. In multivariate analyses, the odds of having postpartum depression was increased more than seven-fold in the presence of conflict with partner (OR = 7.52, 95% CI: 2.65–23.13). The association between antepartum and postpartum depression was quite strong but did not reach statistical significance (OR = 3.37, 95% CI: 0.98–11.64).

**Conclusions:**

The high prevalence of significant postnatal depressive symptoms among Kenyan women underscores the need for addressing this public health burden. Depression screening and psychosocial support interventions that address partner conflict resolution should be offered as part of maternal health care.

## Background

Perinatal depression is a public health concern worldwide with research showing a higher prevalence in low resource contexts [1, 2]. In high resource countries prevalence estimates of antepartum depression range from 7 to 15% [3, 4] while in low resource countries estimates ranging from 15 to 25% have been reported [1, 2]. Postpartum depression prevalence estimates also follow a similar pattern with estimates of nearly 10% in high resource countries [5] compared to 20% in low resource countries [1]. Estimates from East Africa are about 20% in the antepartum period [6] and have been shown to range from 6 to 39% during the postpartum period [6, 7]. This wide range has been largely attributed to differences in assessment tools as well as study populations across the studies [1].

Perinatal depression, reflected in both clinical depression and significant depressive symptoms, carries adverse physical and psychological consequences for both the mother and child [8]. Antepartum depression has been linked to higher rates of spontaneous abortion, prolonged labour and operative deliveries in the untreated mother [9–11]. Poor birth outcomes like preterm births and low birth weight are also higher in women with antepartum depression [11–13]. In a recent Kenyan study antepartum depression was linked with pre-term delivery [14]. In the postpartum period, mothers with depression show poor interaction with their infants with negative consequences to their child’s cognitive and physical development [15–17]. Mothers with postpartum depression are more likely to display either intrusive or withdrawn interactive patterns [8]. The intrusive pattern is characterized by hostile affect, while withdrawn mothers are often disengaged and unresponsive. The consequences of these maternal response patterns are higher risk of dysregulated attention and arousal in the infant’s cognitive development that can have adverse effects on affect regulation, learning and intelligence [11–13]. A large longitudinal study in sub Saharan found a 50% increase in new-born illnesses [18]. The effects of maternal depression are not limited to only the infancy stage but have been shown to persist into the toddler and adolescent stages of life. Longitudinal studies have shown higher risk of childhood disorders like ADHD and conduct disorders that may persist on into adolescence among children of depressed mothers [19, 20]. In a Kenyan study, mothers with postnatal depression were more likely to have underweight infants [21].

The prior mental health status of the mother is a strong determinant of postpartum depression. Specifically, a previous history of depression, presence of antepartum depression, experiencing stressful life events during pregnancy and low perceived levels of social support contribute to increased risk for postpartum depression [1, 22, 23]. Other contributors include obstetric complications, single marital status and low income [24]. Recent Kenyan studies have shown an exceptionally high prevalence of depression among mothers who are HIV positive (48%) [25], among pregnant adolescents (58%) [26] and in mothers with malnourished babies (66%) [14].

Identifying risk factors predisposing to postpartum depression is important especially in guiding proper screening for the condition. The authors have done substantial clinical work and research on depression in Kenya [25, 27], and have identified the need for further assessment of risk factors for PPD in this population. Moreover, research has shown that postpartum depression risk factors in low income countries are, to a large extent, influenced by culture [28]. This work is a step towards understanding risk factors for postpartum depression in the cultural context of Kenya. To date most of what we have learned about risk factors for postpartum depression in Sub-Saharan Africa has been generated with cross-sectional studies [29]. Because retrospective report of social and emotional factors is likely to be biased by current mental health status, prospective studies that assess risk factors during pregnancy and then mental health outcome during the postpartum period can yield stronger inferences. In a prior publication we reported univariate associations between postpartum depression and a broad spectrum of antepartum and postpartum risk factors [30]. In this study, we expand on the previous work by assessing multivariable associations with PPD.

The aims of the current study were to estimate the prevalence and incidence of significant postpartum depressive symptoms in a cohort of pregnant women residing in an urban resource poor setting in Nairobi, Kenya and to use multivariate analyses to determine the unique contributions of antenatal depression and selected demographic, psychosocial, and clinical factors to postpartum depression risk.

## Methods

### Study population and setting

Women were recruited from the outpatient waiting areas of maternal and child health (MCH) clinics associated with two major public hospitals (Mathari Teaching and Referral Hospital and Mbagathi District Hospital) in Nairobi, Kenya, between March and December 2014. The maternal and child health clinics offer both antenatal and postnatal care, including family planning and infant immunization. The majority of the women served by these two hospitals are from an urban, resource-poor catchment area.

### Participant recruitment and screening

Women were eligible if they were pregnant, between ages 18 and 49 years, and in their third trimester of pregnancy. Gestational age was verified using the patient attendance card. Since the study had a follow up time point to assess postpartum depression, participating women had to be willing to seek postnatal care at the same facility. Following registration at the MCH clinic, trained study nurses informed all attending women in their third trimester of the ongoing study. After giving an explanation of the study, a written informed consent was obtained from those who met the inclusion criteria.

### Ethics and consent

We obtained approval to carry out the study from the Kenya Medical Research Institute Ethics Review Board. We further got written permissions to carry out the research from the medical superintendents of the 2 facilities. We informed eligible participants that the study participation was voluntary, and information collected during the study would be used solely for the purposes of the study. Willing participants signed a written informed consent after detailed explanation of the study purpose.

### Assessment procedures

Demographic, psychosocial and clinical history data were collected using structured questionnaires during a face-to-face interview. Depression levels were assessed at baseline and again at 6–10 weeks postpartum using the Edinburgh Postpartum Depression Scale (EPDS). Nurses working at the facility administered the structured demographic, psychosocial and clinical questionnaires and the EPDS screening tool. The nurses were trained to conduct the interviews by the first author (LO) as part of a larger study to evaluate the feasibility and acceptability of integrating depression screening with the EPDS into Kenyan MCH clinics.

### Perinatal depression

The Edinburgh Postnatal Depression Scale (EPDS or EDS when used in the antenatal period) is the most widely used scale to screen for antepartum and postpartum depression symptoms in low and middle-income countries [31]. It is a 10-item questionnaire, with each item scored from 0 to 3; total scores range from 0 to 30. The scale has been validated for detection of depression in both antepartum and postpartum samples [32]. Prior to our data collection, the EPDS tool was translated and back translated through a rigorous process into Kiswahili language [33]. A cut off of 13 or more was set as an indication of antepartum depression, and a cut off of 10 or more was used to indicate postpartum depression, as recommended by Murray and Cox in the assessment for minor antepartum depression and minor postpartum depression, respectively [34].

### Factors investigated for association with postpartum depression

Demographic, psychosocial and clinical factors were selected based on a literature review of studies of maternal mental health. Demographic factors included age, marital status, religion, level of education and occupation of both the mother and her partner. Household income was assessed on the basis of the daily household food expenditure.

Psychosocial factors were assessed during the antenatal period using single items created by the authors. These included: relationship with the mother-in-law (*good, not good but can cope, bad and cannot cope, N/A*); conflict with the partner in the previous 12 months (*any verbal or physical, none, N/A*); partner’s help with cooking, cleaning and/or childcare (*any, none, N/A*); and economic stress within the household during the pregnancy (*yes, no*).

Postnatal clinical factors assessed were mode of delivery (vaginal or cesarean), low birth weight, and nursery admission. A binary composite variable consisting of birth complications (yes/no) and birth outcome (baby alive/died) was created, such that 1 = birth complications present and/or mother lost baby, and zero otherwise.

For this analysis, we did not have a primary exposure of interest since our goal was to assess multiple risk factors. However, if we consider the exposure of antenatal depression, we had 72% power to detect a crude odds ratio for prenatal depression of 3.1 comparing women who had PPD and those who did not.

### Statistical analysis

The prevalence and incidence of postpartum depression and their 95% confidence intervals were determined. Incidence was calculated as the number of new cases of depression at the postnatal assessment divided by the number of postpartum person months contributed by all of the participating women. The 95% confidence interval around the incidence estimate was calculated using Fisher’s exact test. Factors associated with postpartum depression were evaluated using multivariate logistic regression. We added variables in three successive steps. In step 1 we entered prenatal depression status and demographic variables; in step 2, we added the psychosocial variables, and in the final step, we added the postnatal clinical variables. All analyses were performed using R version 3.1.2, and significance level evaluated at 5%.

## Results

**Fig. 1:**
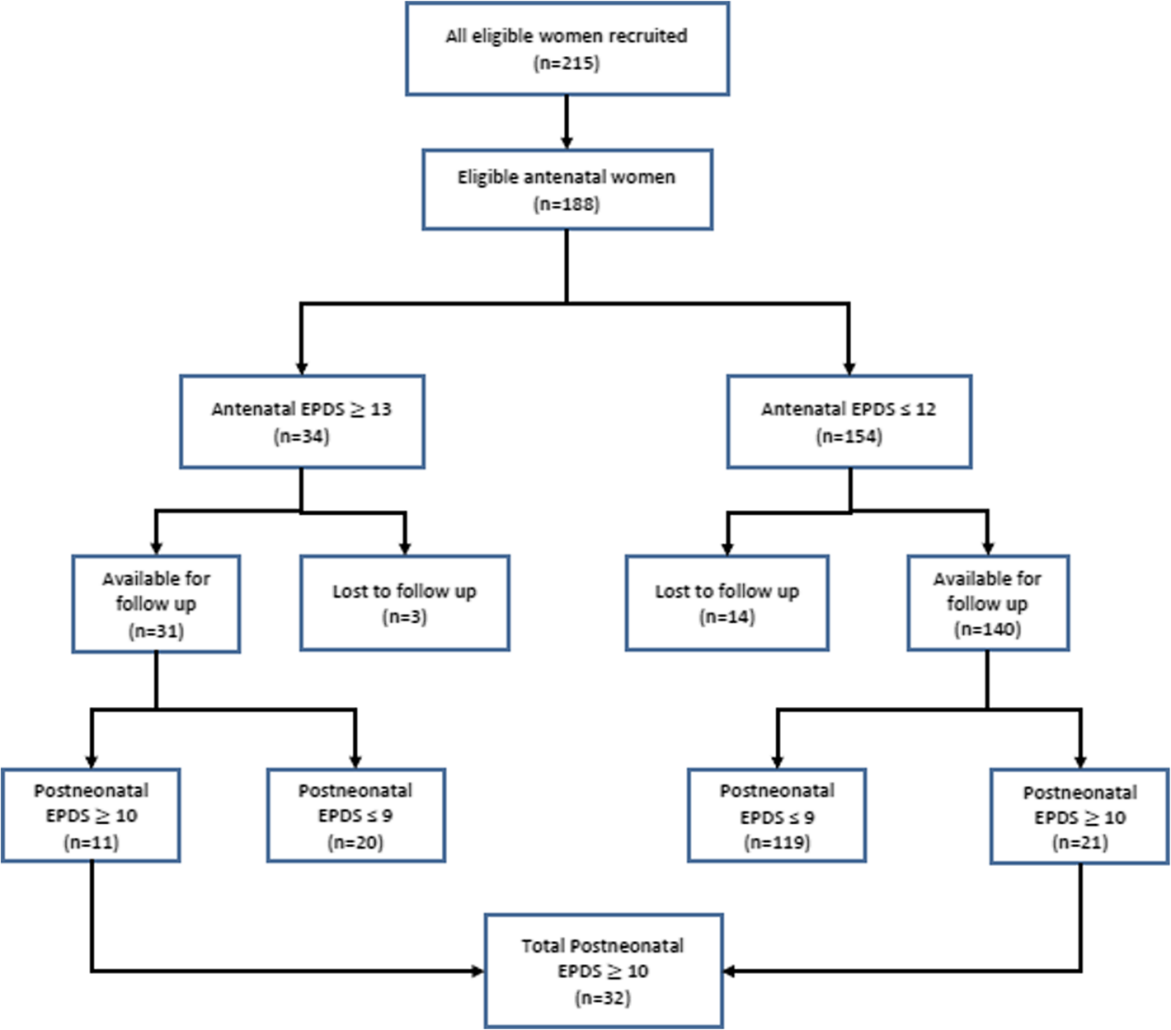
Study profile

Of the 215 women who were eligible for enrollment, seven refused to participate due to time constraints (four women) and the need to obtain permission from their partners before enrollment (three women). An additional 20 women were excluded because they did not plan to continue with postnatal care (including vaccination and family planning) in the same facility. A total of 188 women were included in the study. Seventeen women were lost to follow-up after antenatal assessment, 10 of whom reported to have moved to their rural home and hence not willing to continue as study participants, the rest could not be traced on phone despite 3 attempts to reach them thus 171 were assessed postpartum (Fig. 1).

The prevalence of significant depressive symptoms at 6–10 weeks postpartum was 18.7% (95% CI: 13.3–25.5). The cumulative incidence of postpartum depression (PPD) among the 140 women who did not have antenatal depression was 21 new cases or 15.0% (95% CI: 0.10–0.22).

The distribution of most demographic variables was comparable between those with and without PPD. Most women were married (91% vs. 87% of those with and without PPD, respectively), had at least secondary education (69% vs. 74%), and were Catholic or Protestant (78% vs. 88%). The median age of women in both groups was 25 years. Larger variations were seen in number of children and family income. Of women with PPD, 6% had three or more children, compared to 24% of women without PPD. Of women with PPD, 56% had a monthly family income of Kenyan Shillings (KES) 24,000 or less, compared to 36% of women without PPD (Table 1).

**Table 1:** Demographic Characteristics of Participants

Variable	Postpartum depression	
	Yes (*n* = 32)	No (*n* = 139)
Age in years; median (IQR)	25.5 (22.8, 28.0)	25.0 (22.5, 29.0)
Marital status; n (%)		
Never married/Separated	3 (9)	18 (13)
Married	29 (91)	121 (87)
Highest level of education; n (%)		
≤ Primary	10 (31)	36 (26)
Secondary	17 (53)	62 (45)
Tertiary (college/university)	5 (16)	41 (29)
Religion; n (%)		
Catholic	8 (25)	44 (32)
Protestant	17 (53)	78 (56)
Other	7 (22)	17 (12)
Monthly Family Income in KES; n (%)		
≤ 24,000	18 (56)	50 (36)
> 24,100	14 (44)	89 (64)
Number of children¥		
0–1	10 (31)	54 (39)
2	20 (63)	51 (37)
3–5	2 (6)	34 (24)

¥ = *p*-value < 0.02; *IQR* interquartile range, *KES* Kenyan Shillings (1 USD = 100KES);

number of children including newborn

Of women with and without PPD, 56% and 63%, respectively, reported having a good relationship with their mother-in-law, and 53% and 71% reported that their partners were helping with childcare, cooking, and/or cleaning. Women with PPD were more likely to report having recent economic stress and conflict with partner than those without PPD (53% vs. 30% and 56% vs. 15%, respectively) (Table 2).

**Table 2:** Psychosocial Risk Factors for Postpartum Depression

Variable; n (%)	Postpartum depression	
	Yes (*n* = 32)	No (*n* = 139)
Antepartum depression¥		
Yes	11 (34)	20 (14)
No	21 (66)	119 (86)
Relationship with partner’s mother		
Good	18 (56)	87 (63)
Other*	14 (44)	52 (47)
Economic stress¥		
Yes	17 (53)	42 (30)
No	15 (47)	97 (70)
Conflict with partner**,¥		
Physical/verbal	18 (56)	21 (15)
No conflict	14 (44)	118 (85)
Partner helping with cooking, cleaning and child care		
Yes	17 (53)	99 (71)
No	15 (47)	40 (29)

¥ = *p*-value < 0.02; *Other includes bad or not applicable; **Conflict with partner during previous 12 months

Most women had vaginal delivery (84% and 81% of those with and without PPD, respectively), delivered babies of normal birth weight (88% and 91%), and did not report a history of the baby being unwell (84% and 83%). However, the distribution of reported birth complications and nursery admission between women with and without PPD differed; women with PPD were more likely to report birth complications (34% vs 16%) and nursery admission (16% vs 9%) (Table 3). Among all the risk factors assessed in Tables 1, 2 and 3, only number of children, antepartum depression, economic stress and conflict with partner had statistically significant associations with PPD status.

**Table 3:** Postnatal Clinical Risk Factors for Postpartum Depression

Variable; n (%)	Postpartum depression	
	Yes (*n* = 32)	No (*n* = 139)
Mode of Delivery		
Vaginal	27 (84)	112 (81)
Caesarean Section	5 (16)	27 (19)
Reported birth complications		
Present	11 (34)	22 (16)
Absent	21 (66)	117 (84)
Low birth weight		
Yes	4 (12)	13 (9)
No	28 (88)	126 (91)
Nursery admission		
Yes	5 (16)	12 (9)
No	27 (84)	127 (91)
Birth outcome		
Alive	27 (84)	133 (96)
Baby died	5 (16)	6 (4)

In a multivariate analysis adjusted for demographic variables (education, marital status, family income, parity and maternal age) the estimated odds ratio (OR) of PPD comparing those with and without antepartum depression was 2.83 (95% CI: 1.10–7.34, *p*-value: 0.029). In step 2 when psychosocial risk factors (economic stress, conflict with partner, relationship with partner’s mother and partner helping with childcare) were taken into account, this odds ratio was attenuated to 2.50 (95% CI: 0.79–7.77) and was not statistically significant (*p*-value: 0.112). Further adjustment for postnatal clinical factors in step 3 yielded a somewhat stronger, but still not statistically significant, association between PPD and antepartum depression: OR = 3.37 (95% CI: 0.98–11.64, *p*-value = 0.05) (Table 4).

**Table 4:** Logistic regression results for Risk Factors for Postpartum Depression (*N* = 171)

Variable (Reference category)	1^a^	2 ^a^	3 ^a^
Antepartum depression (No)	2.83 (1.09–7.20)*	2.50 (0.79–7.77)	3.37 (0.98–11.64)
Relationship with partner’s mother good (other)		0.69 (0.24–1.95)	0.83 (0.28–2.53)
Economic stress (No)		2.73 (1.02–7.50)*	2.54 (0.89–7.43)
Conflict with partner (No conflict)		7.12 (2.62–20.67)*	7.52 (2.65–23.13)*
Partner helping with child care (No)		0.49 (0.17–1.46)	0.66 (0.20–2.16)
Mode of delivery (C-section)			0.53 (0.15–2.03)
Low birth weight (No)			1.31 (0.19–6.95)
Nursery admission (No)			0.73 (0.11–4.03)
Birth complications/outcome (No/baby alive)			2.71 (0.74–9.93)

^a^OR (95% confidence interval): adjusted for education, marital status, family income, parity and maternal age; *p-value < 0.05

After adjusting for demographic variables and antepartum depression, two psychosocial factors, economic stress and conflict with partner, were found to be positively associated with PPD (OR = 2.73 (95% CI: 1.02–7.50, *p*-value = 0.046) and OR = 7.12 (95% CI: 2.62–20.67, *p*-value < 0.001), respectively). Having a good relationship with the mother-in-law and having a partner who helped with housework and childcare were associated with lower odds of postpartum depression (OR = 0.69 (95% CI: 0.24–1.95, *p*-value = 0.474) and OR = 0.49 (95% CI: 0.17–1.46, *p*-value = 0.197), respectively); however, neither reached statistical significance.

In the final analyses in which postnatal clinical factors were evaluated in addition to demographic variables, psychosocial risk factors and antepartum depression, the odds ratio comparing mothers who had babies with low birth weight to those with normal birth weight was 1.31 (95% CI: 0.19–6.95, *p*-value = 0.764). Similarly, the odds ratio of PPD comparing mothers who had birth complications or death of baby at birth to those whose babies were alive at birth and had no birth complications was 2.71 (95% CI:0.74–9.93, *p*-value = 0.127). On the contrary, vaginal delivery and nursery admission were associated with lower odds of PPD (OR = 0.53 (95% CI: 0.15–2.03) and OR = 0.73 (95% CI: 0.11–4.03), respectively). In these adjusted analyses, none of the birth outcomes was statistically significantly associated with postpartum depression. However, conflict with partner was still positively associated with postpartum depression - the odds ratio comparing women who reported having conflict with partner to those who reported no conflict with partner was 7.52 (95% CI: 2.65–23.13, *p*-value < 0.001).

## Discussion

This study used multivariate analysis to highlight factors associated with postpartum depression in the first prospective study in a sample of Kenyan women recruited from antenatal clinics. The study showed the perinatal period to be a time of high risk for significant depressive symptoms with prevalence of PPD estimated at 18.7% (95% CI: 13.3–25.5). This estimate is within the range of prior prevalence estimates reported in sub-Saharan Africa (6.1–34.7) [6, 35] where poverty, intimate partner violence and HIV infection have been implicated as major contributors [36–39]. In this study, the antepartum risk factors that had the strongest independent associations with postpartum depression included depression during the antepartum period, as well as self-reported conflict with partner and economic stress. However, in multivariate analyses, the magnitude of the effect size for the association between antepartum and postpartum remained large, based on the point estimate, but did not reach statistical significance. Findings from a recent meta-analysis report conducted on over 14,000 women found depression during pregnancy as one of the strongest predictors of postpartum depression [40] . One large cohort study in Ghana similarly reported antenatal depression as the strongest determinant of postnatal depression [41].Similar longitudinal studies that evaluated depression in pregnancy, as well as in the postpartum period, have found a strong association between postpartum depression and both antepartum depression and history of exposure to violence and conflict [29, 42, 43], as well as having a non-supportive husband [22, 32, 35, 38]. In addition to partner conflict, we asked women to evaluate the relationship with their mother in law. While we did not find a statistically significant association between postpartum depression and relationship with mother-in-law, this may be explained by the fact that our study focused on an urban population cohort. Urban settings differ from rural settings where communal living that includes the mother in law is the norm.

Numerous previous studies have shown that strong social support networks evidenced by good interpersonal relationships enhance resilience to stress and hence contribute immensely to protecting the individual from developing depression [44–47]. A supportive husband and a marital relationship with less friction play a part in promoting a strong social support system for the mother. Sustained perceived pressure from economic stress has been found to elevate cortisol levels and hence contribute towards vulnerability for developing depression [48–50]. A study examining the interaction of economic stress and postpartum family support on cortisol levels found women who reported good family support had lower levels of cortisol compared to women with low family support, even those with underlying high perceived economic stress. These findings indicate that having a supportive partner can buffer the detrimental effects of economic stress [51].

Prior research has shown that obstetric and infant-related clinical factors such as intrapartum hemorrhage, prolonged labor and bad outcome of the baby confers a small increased risk of developing postpartum depression [52–54], once demographic and psychosocial factors are adjusted for. However, these clinical factors did not contribute significantly to a higher likelihood of depression among mothers in our study. This further underscores the important contributions of psychosocial factors to postpartum depression risk.

The finding that nearly one in five women reported significant postpartum depressive symptoms in this Kenyan study suggests the need for post-partum depression screening. Our study also suggests that screening for partner conflict during the prenatal period could help to identify pregnant women at risk of later depression. However, screening programs are recommended only in circumstances where women who screen positive have access to follow-up interventions. Rahman et al. (2014) [55] conducted a systematic review of evidence regarding effectiveness of interventions to address common perinatal mental disorders in low and middle income countries. Robust effects on maternal depression were reported in a study of Pakistani mothers with depression who received a multi-modal intervention delivered by trained lay providers that included elements of cognitive behavior therapy, active listening, support for strengthening the mother-infant relationship, and mobilization of family support [56] . Because of the high prevalence of depression in pregnant women and new mothers, the adverse consequences of maternal depression to the health and well-being to mothers and infants, and the availability of screening tools and empirically supported interventions, conditions are rife for prioritizing perinatal depression screening and intervention in low resource settings. Results of our study highlight intervention targets that would be salient for reducing the burden of postpartum depression in Kenyan women.

### Study limitations

One limitation of our study was the small number of women evaluated. While several of the factors that we evaluated, such as the mother having a good relationship with her mother-in-law and her partner helping with child-rearing, were associated with lower odds of postpartum depression, small sample size contributed to wide confidence intervals around odds ratio estimates. In addition, information about the HIV status of mothers was not available – the prevalence of postpartum depression has been shown previously to be high in Kenyan women who are HIV positive [25]. Furthermore, the follow-up assessment for our study was conducted between 6 and 10 weeks after the birth of the infant. However, the risk of postpartum depression remains high up to 24 weeks postpartum. Our study did not include women who had depression onset after 10 weeks.

We administered the EPDS which has been validated [35] and used extensively worldwide. However, since we conducted our study in 2014, a 9-item perinatal depression screening tool has been developed in Kenya that incorporates local idioms and shows high sensitivity and specificity (0.90/0.90) vis a vis meeting DSM-5 diagnostic criteria for major depression [57]. The authors of this study reported considerably lower sensitivity and specificity (0.70/0.72) for the EPDS at the optimal cutoff. Our study was conducted in an urban setting. Thus, caution is warranted in generalizing our findings to rural areas where access to pre- and post-natal care may be more limited, and social support within the community may be stronger. Finally, as this was part of a larger study to confirm feasibility of integrating EPDS depression screening in maternal and child health clinics, we did not include a concurrent use of a diagnostic tool to confirm depression diagnosis.

## Conclusions

To our knowledge this is the first study to evaluate risk factors for postpartum depression prospectively in an urban cohort of Kenyan women. Our results are consistent with prior research conducted in other settings showing antepartum depression, economic stress, and conflict with partner as predictors of postpartum depression and underscore the need for addressing the public health burden of these interrelated problems. To build upon our study findings, we recommend that more multi-wave cohort studies be conducted in Kenya. Future studies should include longer post-partum follow-up periods and utilize the recently validated Kenyan Perinatal Depression Screen, more refined tools for measuring partner conflict and support, and larger population samples that include women from rural settings.

## Abbreviations

ADHD: Attention Deficit Hyperactivity Disorder
EDS: Edinburgh Depression Scale
EPDS: Edinburgh Postpartum Depression Scale
HIV: Human Immunodeficiency Virus
KES: Kenyan Shillings
MCH: Maternal and Child Health
PPD: Postpartum depression
